# Pilot evaluation of the text4baby mobile health program

**DOI:** 10.1186/1471-2458-12-1031

**Published:** 2012-11-26

**Authors:** William Douglas Evans, Jasmine L Wallace, Jeremy Snider

**Affiliations:** 1 School of Public Health and Health Services, The George Washington University, 2175 K Street, NW, Suite 700, Washington, DC 20037, USA; 2 School of Public Health, University of Washington, 1959 NE Pacific Street, Seattle, WA, 98195, USA

**Keywords:** Text4baby, Pregnancy, Pre-natal health care, Mobile health, Health behavior

## Abstract

**Background:**

Mobile phone technologies for health promotion and disease prevention have evolved rapidly, but few studies have tested the efficacy of mobile health in full-fledged programs. *Text4baby* is an example of mobile health based on behavioral theory, and it delivers text messages to traditionally underserved pregnant women and new mothers to change their health, health care beliefs, practices, and behaviors in order to improve clinical outcomes. The purpose of this pilot evaluation study is to assess the efficacy of this text messaging campaign.

**Methods:**

We conducted a randomized pilot evaluation study. All participants were pregnant women first presenting for care at the Fairfax County, Virginia Health Department. We randomized participants to enroll in *text4baby* and receive usual health care (intervention), or continue simply to receive usual care (control). We then conducted a 24-item survey by telephone of attitudes and behaviors related to *text4baby*. We surveyed participants at baseline, before *text4baby* was delivered to the intervention group, and at follow-up at approximately 28 weeks of baby’s gestational age.

**Results:**

We completed 123 baseline interviews in English and in Spanish. Overall, the sample was predominantly of Hispanic origin (79.7%) with an average age of 27.6 years. We completed 90 follow-up interviews, and achieved a 73% retention rate. We used a logistic generalized estimating equation model to evaluate intervention effects on measured outcomes. We found a significant effect of *text4baby* intervention exposure on increased agreement with the attitude statement “I am prepared to be a new mother” (OR = 2.73, CI = 1.04, 7.18, p = 0.042) between baseline and follow-up. For those who had attained a high school education or greater, we observed a significantly higher overall agreement to attitudes against alcohol consumption during pregnancy (OR = 2.80, CI = 1.13, 6.90, p = 0.026). We also observed a significant improvement of attitudes toward alcohol consumption from baseline to follow-up (OR = 3.57, CI = 1.13 – 11.24, p = 0.029).

**Conclusions:**

This pilot study is the first randomized evaluation of *text4baby*. It is a promising program in that exposure to the text messages was associated with changes in specific beliefs targeted by the messages.

## Background

In recent years, there has been a rapid evolution of mobile phone technologies for use in health promotion and disease prevention. The use of such technologies can allow for dissemination of information to large and varied populations. Innovative mobile intervention approaches used in health promotion include interactive voice response phone calls, use of cell phone applications (apps) and tailored Web-based interventions [1,2]. With the widespread adoption and rapid advancement in the capacities of smartphones (mobile phones with an operating system capable of running applications), more technically advanced interventions, including multiple modes of interactivity and opportunities for engagement with consumers, patients, and beneficiaries of health programs, are now possible [3-5].

While mobile health (mHealth) is rapidly advancing with the advent of more powerful mobile phones, one of the phone’s most basic functions – text messaging – has been the most widely used and studied. Text messaging has been demonstrated to be a potentially powerful tool in effecting behavior change [6]. Recent examples of such prevention and health promotion interventions include smoking cessation, weight loss, depression and sexual health [7-10]. In the past, relatively few studies have been conducted to test the efficacy of mHealth programs as a channel for full-fledged health promotion programs, with most mobile programs focusing on reminder systems or similar limited behavioral cues to action [11]. However, this situation has begun to change, one sign of which is heightened interest in mHealth theory and development of behavior change models that incorporate the unique features of the mobile channel [5,12].

In this paper, we report on a randomized pilot study of audience reactions and utilization of pre-natal and post-partum care promotion text messages delivered by the *text4baby* mHealth program. The text4baby program is designed for low-income women who are pregnant or have recently given birth, and need information and motivation to promote adoption and maintenance of healthy behaviors. This study was conducted exclusively among women who initially present for care at the Fairfax County, Virginia Health Department and then receive treatment from a local health care facility in the county. We randomized participants to enroll in *text4baby* and receive usual health care (intervention), or continue simply to receive usual care (control). We then conducted a behavioral survey of participants by telephone, and also use clinical and health services data collected by Fairfax County, Virginia Health Department and InovaCares clinic as part of their standard health assessments. These data sources were both used to evaluate *text4baby* outcomes.

The overall purpose of this study was to evaluate how well *text4baby* text messages are understood, whether they are trusted and liked, acted upon, and whether recommended behaviors are adopted by the target audience of low-income pregnant women and young mothers. The target population suffers from health disparities due to their socio-economic status and inadequate access to important pre-natal care and health information. Thus, sampling from this population offers an opportunity to gather evidence on the potential of *text4baby* to serve as a model program to help alleviate these disparities. It may also provide insight into the efficacy of the messages and aid the National Healthy Mothers, Healthy Babies Coalition (HMHB) in potential improvements to existing messages and creation of new messages or message delivery systems in the future.

The first specific aim of this study was to assess audience exposure, awareness, and cognitive and affective reactions to *text4baby* messages. The second aim was to identify direct effects of text4baby messages on maternal pre-natal care and related health attitudes, beliefs, and behavioral outcomes, including attending pre-natal care visits, eating a healthy diet, taking vitamins with folic acid, avoiding smoking and alcohol consumption, and related health promoting and risk avoidance behaviors.

## Methods

The evolution from simple reminders and cues to action to more in-depth behavior change programs raises the importance of behavior change theory for mHealth. Recent studies have noted both that the relevance of existing behavioral theory, such as Social Cognitive Theory (SCT) and Theory of Planned Behavior (TPB), need to be examined and new models considered for their applicability to mHealth [5,11,13-15]. There is also a need for evaluation studies based on behavior change theory, as previous mHealth evaluations have not tested the theory-based mechanisms (mediators) of behavior change that have been shown to predict adoption and maintenance of health behaviors in previous research [13]. Evaluation models based on theory need to be developed and tested across multiple subject areas.

*Text4baby* is a text messaging service launched in February 2010 that delivers text messages (http://www.text4baby.org) to pregnant women and new mothers. Text4baby specifically targets traditionally underserved women facing health disparities. The intervention sends texts messages to offer immediate, “just-in-time” tips, with the goal of improving prenatal and postpartum health outcomes [13]. *Text4baby* aims to increase maternal expectations for health outcomes and to promote self-efficacy to utilize the health care system and make informed health care choices, outcomes that may not be regularly achieved in a population facing health disparities and with limited health care access. The program is based on traditional behavioral theories including Social Cognitive Theory (SCT), the Transtheoretical Model (TTM), and the Health Belief Model (HBM) [14,16,17]. Following elements of these theories, *text4baby* seeks to build self-efficacy to successfully utilize health care, improve health literacy, and increase expectations for successful pregnancy and new motherhood. It is designed to build knowledge and skills to manage one’s own health and prevent health risks by avoiding smoking, drinking, receiving recommended immunizations, and avoiding similar behavioral risk factors [18].

**Figure 1:**
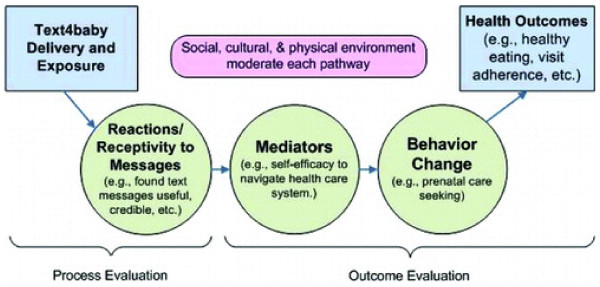
**mHealth conceptual model of behavior change for the Text4baby project. Social cognitive theory and the health belief model used to predict behavior change.** Source: Evans et al, 2012 [13], reprinted by permission of Taylor & Francis (http://www.tandfonline.com).

Figure 1 presents a preliminary model of text4baby that has been used to guide the development of program evaluation strategies.

We evaluated pre-natal text messages (new baby messages were not part of this study) delivered by the *text4baby* program in a randomized pilot study at two clinics that are part of the Fairfax County, Virginia Health Department. Women who initially presented for pre-natal care (first visit) at the Fairfax County, Virginia Health Department and then participate in the InovaCares clinic in Fairfax County were eligible for the study. A majority of the client population spoke Spanish as their primary language. Those who agreed to participate and provide verbal informed consent were randomly assigned into a text4baby exposure or to a no exposure (control) group. Both groups received standard Inova pre-natal counseling and care during study participation. Participants in the text4baby exposure group were directed to enroll in the *text4baby* message service by clinic staff implementing the evaluation study, in order to receive messages for the duration of their pregnancy (or until they dropped out of the program). No incentives were provided for participation in the study. Among womwn who presented at the clinics for prenatal services, 147 agreed to enroll in the study, 123 women responded to the baseline telephone survey (83.7% retention rate). Among the 123 enrollees, 90 completed a follow-up survey, resulting in a 73% retention rate.

### Design and measures

Respondents enrolled in the study completed a 24-item interviewer administered questionnaire developed by the principal investigator. Baseline data collection started in April 2011 and ended in January 2012. Follow-up data collection was completed in April 2012. The survey instrument contained a battery of questions on participant attitudes and behaviors concerning nutrition, smoking and health information-seeking; demographic information such as age, race, ethnicity, education, zip code, marital status and primary language was collected from Fairfax County, Virginia Health Department. The instrument was pre-tested with 7 Spanish-speaking respondents similar to the target population who were debriefed about item comprehension and not included in the subsequent evaluation. The final instrument incorporated revisions based on pretesting. The study protocol was approved as minimal risk research by the George Washington University and Fairfax County, Virginia Health Department institutional review boards on March 15, 2011 (GWU approval number 111047).

The variables for behavioral outcomes were derived from existing, validated instruments, including the Behavioral Risk Factor Surveillance Survey (BRFSS) and National Health and Nutritional Examination Survey (NHANES). An example behavioral outcome variable includes the following: “During the last 3 months, about how many servings of fruit did you have in a day?,” with the following response options [zero servings, 1 or 2 servings per day, 3 or 4 servings per day, 5 or more servings per day or Don’t Know]. Variables specific to attitudes and beliefs were adapted from these same sources and validated instruments used by the investigators in previous research [19,20]. Example attitudes and beliefs variables include the following: “Eating 5 or more fruits and vegetables per day is important to the health of my developing baby,” and “Taking a prenatal vitamin is important to the health of my developing baby,” with the following response options [Strongly Agree, Agree, Disagree, Strongly Disagree, Don’t Know]. Variables specific to the text messages delivered by the *text4baby* mHealth program such as confirmed recall and reactions and receptivity to the messages were adapted by the authors based on validated measures previously published in social marketing evaluation research, including their own work [21,22].

Prior to telephone survey implementation, each new participant was recruited into the evaluation study. The clinical intake staff (mainly nurses), with the assistance of Spanish language translators when necessary, conducted all recruitment activities in-person at the Fairfax County, Virginia Health Department clinic sites. Clinical intake staff requested and drafted a script in conjunction with the research staff to use as a tool to assist in introducing the study, and in following protocol procedures that instructed participants, assigned to the treatment condition, to enroll in the *text4baby* message service after consent. Those who agreed to participate and provide verbal informed consent detailing study procedures were randomly assigned into a *text4baby* exposure or control group based on a randomly generated list of the treatment and control conditions. Clinical intake staff used a pre-generated, randomly ordered list of group assignments in this process. Participants in the *text4baby* exposure group received standard InovaCares pre-natal counseling and care in addition to the *text4baby* messages. Control participants received only standard InovaCares pre-natal counseling and care. No further recruitment efforts were made beyond the individual's visit to the Health Department clinic sites. Individuals who received services but did not consent to the study received no further contact for recruitment.

Participants in the *text4baby* exposure group were instructed to enroll in the *text4baby* message service to receive messages for the duration of their pregnancy (or until they dropped out of the program). These participants enrolled by texting 'BABY' to the short code 511411 (standard *text4baby* enrollment procedure). In addition, they texted the keyword 'CARES' after 'BABY' to signify that they enrolled in the Inova study population (meant to identify them as participants in this study). During implementation, we added a reminder card that stated these instructions to enroll in the *text4baby* service (in English and Spanish), for participants to be able to review the instructions provided by the clinic staff. Upon successful recruitment and enrollment into the *text4baby* message service (participants agreed to study involvement and completed informed consent), their pregnancy due date, zip code, mobile phone number, and the CARES keyword was uploaded to a database managed by Voxiva, Inc. Voxiva is the information technology firm that delivers the automated *text4baby* messages to enrollees. We interviewed participants about their knowledge, attitudes, beliefs, and behaviors concerning pre-natal care, nutrition, physical activity, substance use, vitamins, immunizations, and related health behaviors and risk factors, the primary outcome variables of interest for this study. Participants were followed up, in the same manner, approximately 2–3 months after their initial pre-natal visit (meant to coincide with the participants’ follow-up visit at the InovaCares clinic later in their pregnancy). The purpose of the follow-up interview was to identify differences between the exposure and control groups potentially due to *text4baby* exposure. For *text4baby* exposure group participants, the follow-up interview gathered data about their exposure and reactions to the *text4baby* campaign and its messages in order to determine the effectiveness of *text4baby* messages on maternal, pre-natal care and related health knowledge, attitudes, beliefs, and behavioral outcomes, including attending pre-natal care visits, nutrition, taking vitamins, getting flu shots, avoiding smoking and related health promoting and risk avoidance behaviors.

### Sampling

We randomly sampled from a largely low-income population of women initially seeking pre-natal care from the Fairfax Health Department (who then participated in InovaCares clinic services). The target sample size for the study was 260 participants. This number was estimated based on two factors. First, we estimated a required sample of 130 per comparison group (*text4baby* treatment plus usual care compared to usual care alone) to estimate a 15% difference in repeat (more than one visit) pre-natal care utilization (75% for usual care versus 90% in the *text4baby* exposed group), with a design effect of 1.5. Second, based on enrollment figures for the early months of 2010 (1 year before proposed data collection, same time of year) approximately 500 women presented for care at the same clinics, indicating a sufficient sample from which to recruit during the planned 2 month recruitment period (later extended to approximately 10 months). Recruitment proved difficult for clinical staff operating in their natural setting, preventing us from reaching the target sample size of 260, however, the resulting sample size was adequate for multivariable logistic analyses as confirmed by the consulting statistician on the project. These issues were limitations to the study, as discussed below.

### Data collection procedures

Before data collection initiation, we held an introductory meeting and training sessions at both Fairfax County, Virginia Health Department clinics describing the purpose and importance of the study, study procedures and protocol for clinic staff. Individuals approached and agreeing to enroll in the study were contacted by a George Washington University (GWU) interviewer, supervised by the Principal Investigator (PI), by telephone to complete a pre-exposure, baseline questionnaire. The GWU interviewer was a trained research assistant, fluent in English and Spanish. The telephone-administered survey was approximately 15 min in length; control group participants were also administered the same baseline survey. The GWU interviewer checked faxes received by the 2 clinics on a daily basis during the recruitment period and immediately conducted baseline interviews with newly enrolled participants. Baseline and follow-up interviews were conducted by phone to the participants’ mobile phone (or land-line in cases when participants explicitly requested to carry out the interview on a land-line phone). Enrolled participant names were provided by the clinics and were used solely to develop communication during the phone interview; names were not recorded in the survey database to help execute confidentiality safeguards outlined in the study protocol. At follow-up, we used the same behavioral survey instrument with the addition of a short battery of questions about exposure and reactions to the T4B campaign and its messages.

### Data analysis

We used multilevel logistic regression to construct separate models for each of the attitudes, beliefs, and behavioral outcomes (i.e. fruit and vegetable consumption). Utilizing an interaction term, we estimated the odds of positive change over time in response to each of the behavioral outcome variables as a function of *text4baby* text message exposure, and also as a function of educational status (dichotomized as less than high-school versus high-school or greater). Education status is an important socio-demographic variable that has been shown to moderate effects of health communication interventions such as the one tested in this study and was included as an effect modifier a priori [22,23]. We modeled these outcomes using a generalized estimating equation (GEE) with a multivariable logistic regression model, which allows us to assess population-averaged effects of our predictors, while accounting for correlation of responses within individuals from baseline to follow-up. Participant, age and marital status were included as covariates in each model. Robust sandwich estimators were used to compute standard errors, and an independence working model for correlation was utilized for the generalized estimating equation [24]. Stata Version IC 12.1 (College Station, Texas) was used for the analysis.

The GEE model specification may be expressed in the following formula

(1) $$\begin{array}{l} \text{log}\left[\pi_{\mathrm{ij}}\right]=\beta_{0}+\beta_{1}\text{tim}e_{\mathrm{ij}}+\beta_{2}\text{int}erv_{i}+\gamma_{1}\text{tim}e_{\mathrm{ij}}\mathrm{int}erv_{i}+\beta_{3}educ_{i}\text{tim}e_{\mathrm{ij}}+\\ \beta_{4}\text{angel}20+i\beta_{5}\text{ageg}35_{i}+\beta_{6}\text{marita}l_{i} \end{array}$$

where i = between-subject value, j = within-subject, between-measurement value, time = indicator of pre- or post- measurement value for each subject (1/0), interv = exposure to text-4-baby intervention (1/0), educ = high-school or greater education (1/0), agel20 = maternal age of < 20 years at pre-intervention measurement (1/0), ageg35 = maternal age of > 35 years at pre-intervention measurement (1/0), marital = marital status at pre-intervention measurement (1/0).

For missing data, we excluded cases where we lacked complete outcome data for analysis. We ran a *t*-test to compare covariates, including socio-demographic and other variables used in our regressions, between cases with and without missing data to verify whether or not data were missing completely at random. Non-response dependent on covariates was adjusted for in the models.

## Results

We completed 123 baseline interviews in English and in Spanish – 29 (23.58%) and 94 (76.42%) interviews, respectively. The characteristics of the sample at baseline are shown in Table 1. Overall, the sample was predominantly of Hispanic origin (79.7%) with an average age of 27.6 years. Slightly less than half of respondents (43.4%) reported currently attending school or working outside of the home. Over 70% of the respondents were single/never married, or were part of an unmarried couple relationship. The majority of the sample (58.5%) had completed at least a high school education. We conducted bivariate analysis to examine whether there were differences in socio-demographics based on language (English vs. Spanish) and the results indicate statistically significant differences in race, ethnicity, marital status and education, and having ever gone online to search for pre-natal care information (p <0.05). There were no statistically significant differences in percent ever participating in the WIC program or in percent currently in school/working outside the home by language. We completed 90 follow-up interviews, and achieved a 73% retention rate. Comparison of the baseline and follow-up sample revealed statistically significant differences between the percent of participants reporting having ever participated in the WIC program 75.6% [CI 95: 0.679, 0.833] vs. 86.7% [CI 95: 0.795, 0.938], *p* = 0.045; and between the percent of participants reporting currently working or being in school at 43.4% [CI 95: 0.345, 0.523] vs. 25.6% [CI 95: 0.524, 0.347], *p* = 0.007. We will discuss these as possible study limitations later on in the paper.

**Table 1:** Sample descriptive statistics

**Variables**	**n**	**Mean/%**
Total Study Participants	123	
Age	123	27.6
Age group		
<20	13	10.6
20-34	91	74.0
35+	19	15.5
Race		
White	63	51.2
Black/African-American	10	8.1
Asian	7	5.70
Other/Not Stated	43	35.0
Ethnicity		
Hispanic	98	79.7
Not Hispanic/Not Stated	25	20.3
Marital Status		
Single/Never Married	42	34.2
Unmarried Couple	45	36.6
Married	31	25.2
Separated & Divorced/Annulled	5	4.1
Education		
Less than High School	20	16.3
Some High School	16	13.0
High School Graduate	57	46.3
Some College	8	6.5
College Graduate	7	5.7
None Stated	15	12.2
†Ever Participated in WIC Program	93	75.6
†Currently in School or Working Outside the Home	53	43.4
†Ever Gone Online to Search for Pre-natal care Information	44	35.8

^† ^These data were obtained from the evaluation survey. All other data in Table 1 were derived from clinic records.

Table 2 compares the baseline and follow-up outcome variables used to evaluate the effects of exposure to text4baby messages (intervention) on selected pre-natal attitudes and behaviors. As measured by the attitudinal survey items, the data indicate non-significant increases in strongly agreeing with the selected pre-natal attitudes. For behaviors, there was a slight increase (5%) in the percent of participants reporting having gone online to search for pre-natal care information. Similarly, a very small number of respondents reported consuming alcoholic beverages after finding out about their pregnancy (3.5%) at baseline with a decrease to 1.1% at follow-up. Reported consumption of 3 or more servings of fruit a day increased by 3%. Respondents who reported having smoked in the last 30 days decreased from 5.8% to 1.2% at follow-up, which was significant at the *p* < .10 level (*p* < .098).

**Table 2:** Bivariate pre-post comparison of measured outcome variables by treatment group

	**Baseline sample (n = 86)**				**Follow-up sample (n = 86)**				
**Attitudes, %strongly agree**	**Mean (%)**	**95% CI**	**Control n = 38 (%)**	**Text4baby n = 48 (%)**	**Mean (%)**	**95% CI**	**Control n = 38 (%)**	**Text4baby n = 48 (%)**	P- value*
Eating 5 or more fruits and vegetables per day is important to the health of my developing baby	62.79	51.70, 72.98	71.05	56.25	54.65	43.55, 65.42	63.16	47.92	0.281
Taking a prenatal vitamin is important to the health of my developing baby	62.79	51.70, 72.98	65.79	60.42	51.16	40.14, 62.10	57.89	45.83	0.125
I am prepared to be a new mother	43.02	32.39, 54.15	39.47	45.83	34.88	24.92, 45.92	42.11	29.17	0.276
If I visit my health care provider on a regular basis, I will be a healthy new mother	48.24	37.26, 59.34	47.37	48.94	47.62	36.60, 58.81	54.05	42.55	0.937
If I visit my health care provider on a regular basis, my baby will be healthy	44.17	33.48, 55.30	47.37	41.67	45.35	34.58, 56.45	52.63	39.58	0.879
Smoking will harm the health of my developing baby	53.49	42.41, 64.32	55.26	52.08	47.67	36.79, 58.73	50.00	45.83	0.449
Drinking alcohol will harm the health of my developing baby	52.33	41.27, 63.21	55.26	50.00	44.18	33.48, 55.30	39.47	47.92	0.288
Taking prenatal vitamins will improve the health of my developing baby	47.67	36.79, 58.73	55.26	41.67	46.51	35.68, 57.59	50.00	43.75	0.880
**Behaviors**									
Have you ever gone online to search for pre-natal care information?	31.40	21.81, 42.30	31.58	31.25	36.14	25.88, 47.43	32.43	39.13	0.517
In last 30 days, did you smoke?	5.81	1.91, 13.05	5.26	6.25	1.16	0.03, 6.31	2.63	0.00	0.098
Since you found out about your pregnancy, have you consumed alcoholic beverages?	3.53	0.73, 9.97	2.63	4.26	1.18	0.03, 6.38	2.70	0.00	0.314
Ate 3 or more servings of fruit a day	59.30	48.17, 69.78	63.16	56.25	62.35	51.18, 72.64	67.57	58.33	0.685
Ate 3 or more servings of vegetables a day	36.05	25.97, 47.12	34.21	37.50	34.12	24.18, 45.20	40.54	29.17	0.793

* P-value presented represents the difference between the baseline and follow-up sample means.

Table 3 summarizes the results of the logistic generalized estimating equation model for intervention and education on measured outcomes. We found a significant effect of the exposure to the text4baby intervention on increased agreement with the attitude statement “I am prepared to be a new mother” (OR = 2.73, CI = 1.04, 7.18, p = 0.042) between baseline and follow-up. For those who had attained a high school education or greater, we observed a significantly higher overall agreement to attitudes against alcohol consumption during pregnancy (OR = 2.80, CI = 1.13, 11.24, p = 0.026). We also observed a significant effect on improvement of attitudes toward alcohol consumption from baseline to follow up among those with a HS education or greater (OR = 3.57, CI = 1.13 – 11.24, p = 0.029). While not achieving significance at the *p* < .05 level, we observed several indications, at the *p* < 0.10 level of education status’ association with improvements in several other beliefs - including fruit & vegetable consumption, taking prenatal vitamins, understanding the effects of smoking, and the importance of visiting a health care provider.

**Table 3:** Effects of text4baby and co-variates on improvements in outcome variables from baseline to follow-up

**OR, (95% CI) p-value**	**Eating 5 or more fruits and vegetables per day is important to the health of my developing baby**	**Taking a prenatal vitamin is important to the health of my developing baby**	**I am prepared to be a new mother**	**If I visit my health care provider on a regular basis, I will be a healthy new mother**	**If I visit my health care provider on a regular basis, my baby will be healthy**	**Smoking will harm the health of my developing baby**	**Drinking alcohol will harm the health of my developing baby**	**Taking prenatal vitamins will improve the health of my developing baby**
Effect of text4baby on improvement in agreement	1.04 (0.40-2.73), p = 0.928	1.46 (0.52-4.14), p = 0.474	**2.73 (1.04-7.18), p = 0.042**	1.87 (0.69-5.08), p = 0.221	1.47 (0.56-3.84), p = 0.432	1.17 (0.41-3.36), p = 0.757	0.65 (0.21-2.02), p = 0.457	0.75 (0.32-1.79), p = 0.529
Effect of HS > education on overall agreement	1.61 (0.66-3.91), p = 0.293	*2.25 (0.90-5.65), p = 0.084*	1.99 (0.75-5.31),p = 0.168	*2.29 (0.89-5.88), p = 0.084*	1.84 (0.734-4.62), p = 0.193	2.09 (0.85-5.14), p = 0.106	**2.80 (1.13-6.90) p = 0.026**	*2.44 (1.00-5.95), p = 0.051*
Effect of HS > education on improvement in agreement	*2.51 (0.96-6.58), p = 0.062*	2.38 (0.84-6.74), p = 0.102	2.11 (0.77-5.77), p = 0.144	1.50 (0.56-4.04), p = 0.418	1.80 (0.71-4.57), p = 0.214	*2.51 (0.89-7.03), p = 0.081*	**3.57 (1.13-11.24), p = 0.029**	1.62 (0.67-3.90), p = 0.281

## Discussion

Text4baby is an innovative mHealth program that has grown rapidly. Since its launch in February 2010, nearly 400,000 individuals had enrolled in the service at the time of this writing [25]. This widespread adoption suggests that the program has broad appeal and may represent a valuable health promotion model in the area of maternal and child health. It also raises the question of how effective such a program may be in changing health behavior and also how applicable it may be to other health domains.

This pilot study examined short-term effects of text4baby on attitudes and beliefs targeted by the program, and on immediate health promoting actions pregnant women may take as a result of receiving the messages. Text4baby follows a theory of behavior change illustrated in our conceptual model (Figure 1) that represents a combination of principles from SCT, HBM and the TTM. Thus this evaluation is one step toward validating a new theoretical approach to mHealth programs, one that calls for additional research and theoretical investigation in the field. Previous communication research suggests that targeted health communications delivered using validating messaging strategies may, by themselves, have small but statistically significant effects on subsequent health cognitions and behavior [26,27]. The theory behind text4baby, then, is that beliefs targeted by the program’s text messages will have beneficial effects on those specific beliefs, which in turn will be associated with improvements related to health behaviors. This study examined the relationship between text4baby message exposure and beliefs as immediate program outcomes.

Overall, we found that text4baby exposure indeed was associated with an improvement in one important belief targeted by the messaging. Namely, mothers in the text4baby arm were nearly three times (OR = 2.73) more likely to believe that they were prepared to be new mothers compared to those in the no exposure control group. This may reflect the cumulative effect of multiple messages on a range of topics and the specific focus of those messages on being prepared for the challenges of pregnancy and motherhood and importance of being proactive to maintain good health. However, we did not observe any other effects of the intervention exposure on targeted beliefs.

Additionally, we found a strong effect of education level both on overall agreement (regardless of time) and a pre-post intervention effect on the belief that drinking alcohol during pregnancy will harm the unborn baby. Participants with a high school education or greater were more likely to hold this belief and, while only marginally significant, we observed several other effects of higher levels of education on beliefs targeted by the text4baby messages, as noted above. This may reflect the importance of literacy and comprehension on message effectiveness. Women who are more educated may be better able to process and make informed decisions as a result of text4baby messages. This suggests the potential importance of health literacy in mHealth interventions. This hypothesis should be explored in future studies.

This pilot study had some limitations that deserve attention. Limitations of the study include execution in a natural setting. Although precautions were taken to minimize contamination of subjects in terms of promotion of the text4baby program such as posters in the clinic, some control participants may have been exposed to the program due to its national popularity or through interaction with friends or family members enrolled to receive text messages. However, we have no direct evidence that this took place. In addition, the threat of selection bias is present since it is possible that women who wanted to enroll in the study may have been more motivated than others, or felt the need for more resources than other women.

Another limitation to the study was that we observed differences in the baseline versus follow-up samples due to attrition, with more WIC participants and fewer employed or in school at follow-up. This suggests a more economically disadvantaged sample at follow-up. While these differences should be treated with caution, one explanation is that participants may be at home as they prepare to give birth and may be more in need of WIC resources.

Another limitation was a smaller sample size than planned due to difficulty with recruitment. Anecdotally, reports from clinic staff who met with eligible women found that some had misgivings about enrolling in a service that involved providing mobile phone and other personal information such as their baby’s due date. This may be related to the fact that most women presenting for care were recent immigrants and may not have had complete immigration documentation, making the sharing of personal information seem potentially risky. Clinical intake staff also cited reasons for refusing to participate in the study, such as clients not having their own cell phone but rather using a spouses’ or partner’s phone, being concerned about using cell phone minutes to complete the baseline survey, and the requirement to complete a follow up.

As a result of the smaller than planned sample, we have relatively low statistical power. This reduces our ability to detect potential significant differences from baseline to follow up and between conditions and resulted in relatively wide confidence intervals for observed significant results. However, our sample was sufficient to conduct the planned statistical analyses and this limitation should be considered in the context of the study’s purpose as a pilot evaluation of text4baby. Despite limitations of the sample, the retention rate of 73% from baseline to follow-up was reasonable for a study with an economically disadvantaged sample of primarily non-English speaking immigrants [28].

## Conclusion

This pilot experimental study found that text4baby participation improved a central belief targeted by the campaign – that the pregnant woman receiving text messages was prepared for motherhood. Education was an important factor in reaction to the text messages, suggesting the importance of health literacy in mHealth programs. Future research should examine the differential effects of messages with higher and lower levels of health literacy using experimental methods. The dimension of ‘preparation for motherhood’ should be examined in more depth in future text4baby studies. In particular, ‘preparation’ may reflect certain underlying factors that can be targeted more specifically in future messaging, and this hypothesis should be studied. Finally, large-scale randomized controlled trials should examine the potential for mediated relationships between effects of text4baby and other mHealth programs on health cognitions and subsequent behavior change over time.

## Competing interests

The authors affirm that they have no competing of financial or other interests related to this study.

## Authors’ contributions

WDE was the principal investigator of the study, conceptualized the manuscript and analysis, and wrote sections of the main narrative. JLW managed day-to-day study operations, assisted in analysis, and wrote sections of the main narrative. JS conducted multivariable analysis and wrote sections of the main narrative.

## Pre-publication history

The pre-publication history for this paper can be accessed here:

http://www.biomedcentral.com/1471-2458/12/1031/prepub
